# Comparison of Maximum Intensity Projection and Volume Rendering in Detecting Pulmonary Nodules on Multidetector Computed Tomography

**DOI:** 10.7759/cureus.14025

**Published:** 2021-03-21

**Authors:** Muhammad Qasim Naeem, Jaideep Darira, Muhammad Saad Ahmed, Kamran Hamid, Muhammad Ali, Muhammad Kashif Shazlee

**Affiliations:** 1 Diagnostic Radiology, Dr. Ziauddin Hospital, Karachi, PAK

**Keywords:** maximum intensity projection, volume rendering, mip, vr, pulmonary nodules, low-density nodule

## Abstract

Introduction

Lung cancer is the most common cancer overall, and the foremost cause of cancer-related mortality. Almost all lung cancers evolve from pulmonary nodules. As multidetector CT (MDCT) scanners are now widely available, there is an increased rate of detection of pulmonary nodules. It is of utmost importance to evaluate pulmonary nodules to rule out the possibility of neoplastic diseases. With advancements in technology, there are various manual and automatic analytic software providing a wide range of post-processing techniques. Maximum intensity projection (MIP) and volume rendering (VR) techniques have been analyzed previously regarding pulmonary nodules but there is a scarcity of data in terms of low-density nodules. This study aims to delineate the comparison and supremacy of both techniques in terms of low-density nodules.

Methodology

The current prospective study was conducted from June 2019 to June 2020 in the Radiology Department at Dr. Ziauddin Hospital, Karachi. Chest CT scans were performed on 16 slice MDCT (Alexion 16 Multi-slice, Toshiba Medical System Corporation, Houston, TX). A consultant radiologist of six years experience and a postgraduate trainee of three years experience analyzed each patient on a workstation (Vitrea 6.2.0, Vital Images, Minnetonka, MN). SPSS 23.0 (SPSS Inc., Chicago, IL) was incorporated for data analysis. Data were expressed in the median and interquartile range (IQR). Data collected for this study were analyzed using analyzing the median difference in nodule count using Wilcoxon’s signed-rank test. A p-value of <0.05 was considered significant.

Results

After informed consent, 236 patients were recruited for the study. MIP outperformed VR in terms of nodule detection and low-density nodules at each evaluated slab thicknesses (p<0.001). A 10-mm MIP was superior to all other techniques in terms of detection of pulmonary nodules and low-density nodules (p<0.001). MIP was also considered an easier technique as there was excellent inter-rater reliability and agreement.

Conclusion

This study is robust evidence regarding the supremacy of MIP. MIP outperformed VR on every slab thicknesses. The 10-mm MIP technique was superior to all others evaluated and was recorded to be an easier analyzing technique.

## Introduction

According to World Health Organization, lung cancer is among the most frequent cancer overall and is the noteworthy cause of cancer-related mortality [1,2]. The earliest manifestation of lung cancer is pulmonary nodule [3]. With the wide availability and an increased ratio of chest CT scans, pulmonary nodules are being increasingly detected [3]. However, a wide range of diseases has associated pulmonary nodules, such as infectious, inflammatory as well as neoplastic diseases. Early detection of a pulmonary nodule, as well as its characteristic features such as site, size, and density, needs to be recorded for making a diagnosis [4]. Post-processing techniques were invented to reduce the scanning time, improve the efficiency, and reduce fatigue of the radiologist [4,5]. During routine reporting, a number of studies have established the advantage of post-processing 3D CT techniques, such as maximum intensity projection (MIP) and volume rendering (VR) in the detection and assessment of pulmonary nodules [3-8].

In MIP, voxels having the highest attenuation are projected along lines through the volume of a given dataset [9]. In contrast to MIP, final images in VR are obtained by assigning every tissue a shade and opacity, thus every voxel displays the shade and opacity based upon the proportions of different tissues present, their assigned shades, and opacities [9]. This helps in the preservation of the actual size of the nodule [4].

We evaluated MIP and VR on different slab thicknesses for detecting pulmonary nodules. Our objective of the study was to compare MIP and VR on slab thicknesses of 4-mm, 7-mm, and 10-mm in detecting low-density pulmonary nodules. To the best of our knowledge, very fewer studies have been performed in the past.

## Materials and methods

Research design

This prospective study was conducted from June 2019 to June 2020 in the Radiology Department at Dr. Ziauddin Hospital, Karachi. A favorable ethical approval from the Institutional Research Review Committee was obtained. The study participants were provided a patient information sheet. After written informed consent, all patients of age more than 18 years were consecutively sampled who were referred to our institute for chest CT scan, irrespective of gender. By keeping the sensitivity of 86%, the specificity of 82%, with a 20% prevalence of pulmonary nodules and keeping precision at 10% using an online sample size calculator for sensitivity and specificity studies, a sample size of 236 was estimated which was sufficient for both sensitivity and specificity calculation in our study. Datasets with breathing artefacts, consolidation, pulmonary fibrosis, nodules count >20 in each lung, and pneumoconiosis were excluded from the study.

Image acquisition

A 16-slice multidetector CT scanner (Alexion 16 Multi-slice, Toshiba Medical System Corporation) was used for scanning the chest in the craniocaudal axis from the apices of the lung to the upper abdomen. Scanned data were obtained in helical mode. The acquisition parameters were: tube voltage, 90-135 ㎸ (adjusted according to weight of the patient); collimation, 16 × 1; gantry rotation, 0.75 sec; pitch, 1.0; slice thickness, 1-mm; reconstruction kernel, FC 50-52; and reconstruction increment, 0.6-mm. The required output of tube current-time product was adjusted automatically according to the patient by in-built Toshiba’s Adaptive Iterative Dose Reduction (AIDR) technology. No intravenous contrast was used. Appropriate instructions were conveyed to the patient for breath-holding to minimize breathing artefacts.

Image analysis

Images were analyzed using image viewing software (Vitrea 6.2.0, Vital Images) through a cinematic viewing approach. Data for different characteristics of pulmonary nodules were recorded. Each scan was interpreted by two radiologists independently, one having experience of six years and a postgraduate trainee of three years’ experience, respectively. The software was used to reconstruct MIP and VR images. Window level of −400 HU and window width of 1600 HU were set for analysis of lung nodules on MIP technique, while VR images were evaluated at −500 HU window level and width of −1500 HU. Opacity for VR images was kept at 50%. Both radiologists analyzed each scan six times using the following method: MIP and VR on slab thicknesses of 4-, 7-, and 10-mm, respectively. Larger slab thicknesses were not used because of overlapping of structures and partial volume artefact. Although the analysis was done in cinematic axial approach at times, doubtful nodules were confirmed after seeing the multiple planes. Nodules characteristics such as size and density were evaluated on both post-processing techniques (MIP and VR). Classification of nodules was done on sizes of <6-mm and >6-mm. The density of nodules was categorized as low if HU ≤ 100 and high if HU > 100. Both readers were unaware of the number of pulmonary nodules in each patient. MIP and VR images were analyzed separately with a minimum gap of at least one week to reduce memory effects.

Data analysis

Statistical analysis was performed using SPSS (IBM SPSS Statistics 23.0). Data were expressed in the median and interquartile range (IQR). Data collected for this study were analyzed using analyzing the median difference in nodule count using Wilcoxon’s signed-rank test. A p-value of <0.05 was considered significant. Intraclass correlation coefficient estimates (for interrater reliability) and their 95% confidence intervals were calculated using SPSS statistical package version 23 (SPSS Inc., Chicago, IL) based on a mean-rating (k = 3), absolute-agreement, two-way mixed-effects model. The agreement was classified using a grading system: values down from 0.5 indicate poor reliability, values ranging from 0.5 to 0.75 stipulate moderate reliability, values range 0.75-0.9 stipulate good reliability, and values exceeding 0.90 stipulate excellent reliability [10].

## Results

Two hundred and thirty six patients CT were examined; using MIP technique, more pulmonary nodules were observed (slab thickness 4-mm, n=2028; 7-mm, n=2423; 10-mm, n=2499) as compared to VR technique (slab thickness 4-mm, n= 1693; 7-mm, n= 2081; 10-mm, n= 2169), respectively. Low density nodules were also detected more on MIP (slab thickness 4-mm, n=138; 7-mm, n=192; 10-mm, n=215) than on VR (slab thickness 4-mm, n=67; 7-mm, n=123; 10-mm, n=159).

Interclass correlation coefficient for the calculation of 4-mm MIP was 0.99 (95% CI: 0.98-0.99; p<0.001) 0.99 (95% CI: 0.991-0.999; p<0.001) for 7-mm MIP, and 0.99 (95% CI: 0.98-0.99; p<0.001) for 10-mm MIP which indicated excellent inter-rater reliability and agreement. While, interclass correlation coefficient for the calculation of 4-mm VR was 0.81 (95% CI: 0.688-0.872; p<0.001), 0.89 (95% CI: 0.821-0.932; p<0.001) for 7-mm VR, and 0.91 (95% CI: 0.875-0.938; p<0.001) for 10-mm VR which indicated good to excellent inter-rater reliability and agreement.

## Discussion

As the availability of MDCT has widely grown, the detection of pulmonary nodules has increased. Post-processing software provides various reading techniques such as average, MIP, MinIP, and VR. Recent technical advancements based on artificial intelligence, i.e., computer-aided detection have also shown promising results [11]. However, there is an increased ratio of false-positive results [11,12], recorded to be 33% in one study [11]. Moreover, these software programs are not widely available in developing countries. Any size of lung nodule is important in cases of known malignancy [4]. In the past, several studies established that images analyzed through MIP and VR have superior detection of pulmonary nodules over traditional axial images [4-6,8,13-17]. However, to the best of our knowledge, there is only a single study that has compared MIP and VR in terms of nodule detection with respect to the density at various slab thicknesses [4]. Diederich et al. deduced that 15-mm MIP was superior to any other technique used in their study, however, they had reconstructed datasets by 5- and 10-mm collimation and they did not evaluate nodule density [15]. With advancements in computed tomography, reconstruction with narrower collimations was possible. Kawel et al. compared MIP and VR on 5-, 8-, and 11-mm collimation and inferred that 8-mm MIP outperformed all the included slab thicknesses, in terms of nodule detection [6]. Sharma et al. compared VR and MIP on different slab thicknesses, and inferred 11-mm MIP and VR to be slightly superior in terms of nodule counts and 11-mm MIP to be superior overall [4]; Yoneda et al. assessed nodule detection on 15-mm MIP and VR and draw a similar result [17]. Peloschek et al. compared VR and MIP on fixed slab thickness of 7-mm and deduced that VR was superior to MIP [5].

We found that number of nodules were detected more on the MIP technique than VR technique for each of the observer, and there was a statistically significant difference between these two techniques at every slab thickness, p-value < 0.001 (Table 1). This in fact can be thought that readers were more accustomed to MIP technique than the VR technique, however, this was also recorded that less experienced observer had also detected more nodules on MIP images than VR images, concluding MIP an easier analyzing technique. Our results also show that 10-mm MIP surpassed all other slab thicknesses used; however, there was no statistical significance between 10-mm and 7-mm MIP as both equally detected nearly the same number of nodules (Table 1).

**Table 1:** Correlation of maximum intensity projection and volume rendering at mentioned slab thicknesses with respect to nodule count.

Slab thicknesses	Number of nodules detected		Median		IQR		P-value
	MIP	VR	MIP	VR	MIP	VR	
4-mm	2028	1693	7.0	6.0	8	7	<0.001
7-mm	2423	2081	9.0	8.0	11	9	<0.001
10-mm	2499	2169	10.0	9.0	11	9	<0.001

The size was another criteria, we evaluated supremacy of these two techniques in only two categories, i.e., <6-mm and >6-mm, as there are plentiful data in the previous studies [4-8]. Sharma et al. reported that MIP was superior to VR only in nodules less than 6-mm, while there was no statistical significance between MIP and VR at nodule sizes of 7-10-mm [4]. Peloschek et al. [5] found VR to be superior over MIP in nodule sizes <10-mm while using a fixed 7-mm slab thickness, whereas Kawel et al. [6] recorded although MIP was superior overall, there was less difference in detecting nodule of sizes >8-mm. We found that 4- and 7-mm MIP picked more number of nodules of two different categories as compared to the same slab thickness of VR (p<0.001-0.003; Table 2); however, at 10-mm slab thickness, VR was equally effective in detecting nodules of >6-mm size but not for <6-mm size, (p-value = 1).

**Table 2:** Correlation of maximum intensity projection and volume rendering at mentioned slab thicknesses with respect to nodule size.

Slab thickness		Size <6-mm			Size >6-mm		
		MIP	VR	P-value	MIP	VR	P-value
4-mm	Median	6.0	4.0	<0.001	1.0	1.0	<0.001
	IQR	7	5		2	1	
7-mm	Median	8.0	6.0	<0.001	2.0	2.0	0.003
	IQR	9	7		4	2	
10-mm	Median	8.0	6.0	<0.001	2.0	2.0	1
	IQR	9	7		2	2	

We also found that using MIP enhanced detection of low-density nodules, despite the fact that anatomic details are preserved by VR technique [5]. In our study, MIP detected more low-density nodules as compared to VR on every evaluated slab thickness; however, this may be due to the fact that the VR technique uses the opacity value assigned to voxels and is measured in percentages from 0% to 100%. At higher opacity percentages, the object looks bigger than its normal size, and lower opacity percentages make the object smaller. Thus, a high-density structure can be detected more easily than a low-density structure. It is also noteworthy that each software has its own incorporated VR algorithm, which results in image representation variations that can influence the contrast and detection rates of the nodule [9]. In our study, MIP detected more low-density nodules than VR on all provided slab thicknesses (Table 3). Similarly, Sharma et al. recorded that low-density nodules were significantly detected more by MIP than by VR [4].

**Table 3:** Correlation of maximum intensity projection and volume rendering at mentioned slab thicknesses in the detection of low-density nodules.

Slab thickness		Low-density		
		MIP	VR	P-value
4-mm	Median	0.00	0.00	<0.001
	IQR	1	1	
7-mm	Median	0.00	0.00	<0.001
	IQR	2	1	
10-mm	Median	0.00	0.00	<0.001
	IQR	2	1	

Although we did not evaluate reading time for each of the datasets, however, we found that both users were able to report MIP in a lesser time duration than VR, which represents MIP being a user-friendly technique.

There were few limitations of the study. We postulated results on the data recorded by reader A; the other reader was resident for which we evaluated inter-rater agreement only. The excellent inter-rater agreement shows that MIP is easier to interpret and is adjustable according to the reader’s professional experience. Our study also lacks a true reference standard because histopathological proof cannot be obtained, and we do know that only a CT scan can provide nodule characteristics in a non-invasive approach. As the data remains scarce, more work is required to evaluate the role of MIP and VR in the evaluation of low-density nodules.

## Conclusions

This study is robust evidence that the MIP technique is superior to the VR technique in terms of nodule detection as well as detection of low-density nodules. In our study, MIP outperformed VR on each analyzed slab thickness. MIP was also easier to interpret and had an excellent inter-reader agreement. The 10-mm MIP was superior for nodule detection as well as low-density nodules over all the analyzed slab thicknesses.
